# Systematic review of literature regarding the isolation of mesenchymal adult stem cells from the olfactory epithelium

**DOI:** 10.3389/fncel.2026.1735284

**Published:** 2026-03-31

**Authors:** Carlotta Pipolo, Paula La Rubia, Anna Cozzi, Preetha Karki, Alberto Maria Saibene, Daniele Bottai

**Affiliations:** 1 Department of Health Sciences, Otolaryngology Unit, Santi Paolo e Carlo Hospital, Università Degli Studi di Milano, Milan, Italy; 2 Department of Pharmaceutical Sciences Section of Pharmacology and Biosciences, Università Degli Studi di Milano, Milan, Italy

**Keywords:** isolation, nasal biopsy, nasal brushing, neural stem cells, nose, olfactory epithelium, olfactory mucosa, olfactory stem cell culture

## Abstract

**Background:**

The olfactory mucosa has emerged as a promising source of mesenchymal stem cells with neurogenic potential. These cells exhibit neural, glial, and mesenchymal properties, making them attractive candidates for regenerative medicine, particularly in treating neurodegenerative and immunemediated disorders.

**Methods:**

This systematic review analyzed existing literature on the isolation, characterization, and therapeutic applications of olfactory mucosa mesenchymal stem cells. The review assessed variations in isolation techniques, culture conditions, and differentiation potential, as well as preclinical and clinical applications.

**Results:**

Olfactory mucosa mesenchymal stem cells express key neural and mesenchymal markers, including Nestin, SRY-box 2, *Glial Fibrillary Acidic protein*, CD44, and CD105, confirming their multilineage differentiation capacity. Their ability to secrete neurotrophic factors such as Brain-Derived Neurotrophic Factor, Nerve Growth Factor, and Glial Cell Derived Neurotrophic Factor underscores their role in neural repair. While most studies successfully isolated olfactory mucosa mesenchymal stem cells via biopsy, differences in sampling depth and culture media influenced cell yield and growth patterns. Preclinical studies suggest that olfactory mucosa mesenchymal stem cells (OM-MSCs) may represent a promising experimental model for neurological disorders—including Parkinson’s disease, spinal cord injury, schizophrenia, and retinal diseases—although current evidence remains preliminary and translational efficacy has not yet been established. However, challenges remain in standardizing protocols, addressing donor variability, and ensuring clinical safety.

**Conclusion:**

Olfactory mucosa mesenchymal stem cells represent a promising avenue for neurological and regenerative therapies. Despite their potential, further research is needed to optimize isolation techniques, enhance reproducibility, and navigate regulatory hurdles. Collaborative efforts between researchers, clinicians, and regulatory bodies will be essential to translating OM-MSC research into viable clinical applications.

## Introduction

Over the past decades, stem cell research has advanced substantially, with its potential in healthcare and in expanding our understanding of human biology increasingly recognized by the scientific community. Stem cells derived from diverse tissues are being harvested, isolated, and studied, not only as reservoirs for clinical applications but also as models to investigate the cellular processes and signaling pathways that govern tissue function and regeneration, furthermore new approaches such as induced pluripotent stem cells (iPSCs) generation and differentiation, organoid production and CRISP-Cas9 editing changed the research in stem cells not only as therapeutic tools but also as potential models to study human development and disease progression (Bosio et al., 2009; Lee and Son, 2021; Bellon, 2024; Whiteley et al., 2022).

The olfactory neuroepithelium, located within the olfactory cleft and lining the upper nasal septum, dorsal nasal vault, and superior turbinate, performs the initial step of olfaction (Ross and Pawlina, 2006; Pinto, 2011). In mammals, chemosensory input from the olfactory system is essential for survival, feeding, reproduction, and predator avoidance, highlighting its evolutionary importance (Pinto, 2011; Agafonova et al., 2025; Díaz et al., 2013; Costanzo et al., 1992). Although in humans, olfaction plays a less dominant role, it still contributes to nutrition, safety, sensory pleasure, and overall well-being (Pinto, 2011).

In humans, the olfactory mucosa (OM) measures 60–80 μm in thickness, covers ~10 cm^2^, and is composed of a pseudostratified epithelium with basal, supporting, and olfactory receptor cells but lacking goblet cells (Ross and Pawlina, 2006; Patel and Pinto, 2014; Choi and Goldstein, 2018). Cells present in this region include olfactory ensheathing cells (OECs), a specialized glial population within the olfactory system, are among the cells found in this area. They share traits with both Schwann cells and astrocytes, which are linked with the peripheral and central nervous systems, respectively. OECs maintain close contact with the small, unmyelinated axons of olfactory receptor neurons, guiding them from the basal lamina of the epithelium to the olfactory bulb. OECs are also located in regions where other stem cell populations can be found; however, they are not the primary focus of this review, as their relevance is secondary to the stem cell types under discussion (Agafonova et al., 2025).

A hallmark of the olfactory system is its lifelong neurogenesis. Because olfactory sensory neurons (OSNs) are exposed to environmental insults (including pathogens, toxins, and trauma) and survive for only a few months before apoptosis, they must be continuously replaced by newly generated neurons derived from resident Neural Stem Cells (NSCs) (Mackay-Sim, 2010; Choi and Goldstein, 2018).

The persistence of olfaction throughout life, despite this vulnerability, underscores the regenerative capacity of the olfactory epithelium. This capacity is mediated by stem cells capable of self-renewal and progression through intermediate progenitors to form multiple differentiated cell types, including neurons (Berger et al., 2020; Chen et al., 2019; Durante et al., 2020). Two main populations are found in the basal layer: globose basal cells (GBCs), which actively replenish neurons, and horizontal basal cells (HBCs), quiescent reserve cells that activate after severe epithelial injury (Choi and Goldstein, 2018; Mackay-Sim, 2010; Calof et al., 1998; Carter et al., 2004; Schwob et al., 2017). Another population, olfactory epithelium mesenchymal stem cells (OE-MSCs), resides in the lamina propria. These cells combine mesenchymal and NSCs features, with the ability to differentiate into mesodermal lineages or cross the basement membrane to generate OSNs (Calof et al., 1998; Schwob et al., 2017).

Multiple terms have been described in literature to define OM-derived stem/progenitor populations. In this review, we use the following operational definitions: (1) Neural stem/progenitor cells (NSCs): basal epithelial stem/progenitor cells within the olfactory epithelium (including GBCs and HBCs), primarily generating neuronal and supporting lineages; (2) Olfactory epithelium / olfactory mucosa mesenchymal stem cells (OE-MSCs / OM-MSCs; also referred to as ecto-MSCs): mesenchymal-like stromal cells predominantly located in the lamina propria, typically adherent and expressing MSC markers (e.g., CD73/CD90/CD105) with variable neural/glial markers (Nestin/Sox2/GFAP); (3) Olfactory stem cells (OSCs)/olfactory neurosphere-derived cells: an umbrella term often used for mixed sphere-forming cultures containing epithelial progenitors and stromal components, whose phenotype is strongly protocol-dependent. Where possible, we report the original authors’ terminology but interpret results according to anatomical origin and marker profile to avoid conflating distinct cell populations.

The presence of these diverse stem cell types has been confirmed in both humans and other mammals, suggesting that similar mechanisms of stem cell activity extend across species (Calof et al., 1998; Schwob et al., 2017; Mackay-Sim, 2010). A unique advantage of the olfactory system is its anatomical accessibility in humans and other mammals, permitting direct sampling of NSCs and progenitors. Because these cells share developmental origins with central nervous system neurons and glia, they provide a valuable model for studying neural development and regeneration without invasive brain procedures.

Despite this promise, methods for isolating OM-derived stem cells remain heterogeneous. Most studies have used biopsy, a technique that provides adequate tissue but is invasive and difficult to standardize (Schwob et al., 2017; Lanza et al., 1993; Fletcher et al., 2017). To reduce invasiveness, nasal brushing was introduced more than 30 years ago, yielding high cell numbers with minimal trauma by using nylon-bristled brushes (Jafek et al., 2002; Stokes et al., 2014; Orrú et al., 2014; Féron et al., 1998). However, relatively few studies have adopted this method, limiting comparability across protocols. The objective of this systematic review is to critically evaluate harvesting and culture methods for olfactory mucosa stem cells, highlight methodological differences, and assess how these influence outcomes. Specifically, we examine approaches to sampling, pre-culture processing, and culture conditions, as well as the impact of added growth factors or supplements. We also analyze the reported success of isolations in terms of neuronal lineage differentiation. To our knowledge, no prior systematic review has synthesized these aspects, and we aim to provide a comprehensive framework for future research.

### Materials and methods

### Search strategy

A systematic review was conducted between May 1, 2022, and November 10, 2024, according to the Preferred Reporting Items for Systematic Reviews and Meta-analyses (PRISMA) reporting guidelines (Liberati et al., 2009). A systematic electronic search for studies was conducted that reported research done on mammals in the last 12 years focusing entirely or partly on the isolation of stem cells from the nasal olfactory epithelium in mammals. Only articles in the English language were selected.

We searched PubMed, Scopus, and Embase databases with wide search strategies for Isolation of Stem cells from nasal olfactory epithelium from humans and other mammals, with samples obtained by either brushing or biopsy. The details of our full literature search strategy and the number of unique terms retrieved from each database are available in Table 1.

**Table 1:** Summary of included studies.

Study and year	Type of mammal, age	Sample population	Sample harvesting technique	Detail of preparation of sample for culture
Benítez-King et al. (2011)	Human mean age 27.5 years	8	Nasal brushing	(1) Exfoliation. (2) Tissue washing with antibiotics. (3) Mechanical dissociation. (4) Basic medium. (5) Layer.
Krolewski et al. (2011)	B6.129F1 Mice	N/A	Nasal biopsy	(1) Simple dissection and mincing. (2) Tissue washing with antibiotics. (3) Mechanical dissociation. (4) Complex medium. (5) Spheres.
Toft et al. (2012)	Fischer rats	N/A	Nasal biopsy	(1) Simple dissection and mincing. (2) Tissue washing with antibiotics. (3) Enzymatic and mechanical dissociation. (4) Complex medium. (5) Spheres.
Lindsay et al. (2013)	Human Range 17–70 year; Average male 43.5; Average female 40.3	39	Nasal biopsy	(1) Simple dissection and mincing. (2) Tissue washing with antibiotics. (3) Enzymatic and mechanical dissociation. (4) Complex medium. (5) Spheres.
Stamegna et al. (2014)	Sprague Dawley Rat 10 weeks old	50	Nasal biopsy	(1) Simple dissection and mincing, direct plating without dissociation or with enzymatic and mechanical dissociation. (2) Complex medium. (3) Spheres (dissociated by trypsin).
Thakur et al. (2014)	Albino Wistar Rat	6	Nasal biopsy	(1) Complex dissection and mincing. (2) Complex medium.
Ohnishi et al. (2015)	Human Median 30 ± 11.5 years	5	Nasal biopsy	(1) Simple dissection and mincing Wash the tissue sample. (2) Enzymatic dissociation. (3) Complex medium. (4) Spheres.
Altunbaş et al. (2016)	Canine	N/A	Nasal biopsy	(1) Simple dissection and mincing. (2) Tissue washing with antibiotics. (3) Direct plating of the undissociated tissue in basic medium. (4) Recover of the cells. (5) Plating in complex medium. (6) Spheres.
Ercolin et al. (2016)	Rabbit 3 days old	1	Nasal biopsy	(1) Simple dissection and mincing. (2) Tissue washing with antibiotics. (3) Direct plating of the undissociated tissue in basic medium (15% FBS). (4) Plating in basic medium. After 72 h of incubation remove non-adherent cells. (5) Cultivate adherent cells until 70% confluence in basic medium (15% FBS).
Batioglu-Karaaltin et al. (2016)	Human 18, 27 & 40 years	3	Nasal biopsy	Complex dissection and mincing. Plating in basic medium, refreshed every other day, and cell adhesion is observed after 2 weeks.
Ge et al. (2016)	Human 24–49 years	4	Nasal biopsy	(1) Complex dissection and mincing. (2) Direct plating of the tissue, simple medium was changed every 2 or 4 days. (3) Seven to 8 days after, stem cells will begin to invade the culture dish and after 2–3 weeks. (4) When confluency is reached, passage and transfer the cells to culture flasks. (5) After the first harvesting plate in basic medium added with FGF2 and EGF, medium was added every 2 to 4 days.
Tanos et al. (2017)	Human Median 38.5 ± 26 years	10; 6 culture samples 4 paraffin samples	Nasal endoscopic biopsy	Complex dissection and mincing. Enzymatic dissociation. Plating in basic medium. When adherent cells reached confluency, they were passaged using trypsin. Sphere cells were allowed to grow for 7 days.
Franco et al. (2017)	Humans and Swiss-Webster Mice (5 weeks)	N/A	Nasal brushing in humans: Nasal biopsy in mice:	Exfoliation. Tissue washing with antibiotics. Enzymatic dissociation. Plating in complex medium. Spheres were weekly passaged.
Lu et al. (2017)	Human	20	Nasal Biopsy	(1) Simple dissection and mincing. (2) Tissue washing with antibiotics: (3) Direct tissue plating in basic medium. (4) Layer.
Muniswami et al. (2017)	Rat	10	Nasal biopsy	(1) Simple dissection and mincing. (2) Tissue washing with antibiotics. (3) Enzymatic dissociation. (4) Plating in complex medium. (5) Change the medium every 2 days.
Veron et al. (2018)	6 genera: Mouse, Rat, Rabbit, Sheep, Dog, Horse, Gray mouse lemur, Macaque	Total 34 6 Mice, 6 Rats, 6 Rabbits, 3 Sheep, 6 Dogs, 4 Horses, 2 Gray mouse lemur, & 1 Macaque	Nasal biopsy	(1) Simple dissection and mincing. (2) Tissue washing with antibiotics. (3) Enzymatic dissociation. (4) Plating in complex medium. (5) Change the medium every 2 days.
Li et al. (2018)	Wistar Rats (Embryonic day 17)	N/A	Nasal biopsy	(1) Simple dissection and mincing. (2) Tissue washing with antibiotics. (3) Enzymatic dissociation. (4) Plating in complex medium. (5) Change the medium every 2 days. (6) Neurospheres.
Jiménez-Vaca et al. (2018)	Human 32.5 years old (SEM ± 3.23)	22 healthy adults 10 females 12 males	Nasal brushing	(1) Exfoliation. (2) Tissue washing with antibiotics. (3) Mechanical dissociation. (4) Plating in Complex medium. (5) Spheres.
Bagher et al. (2018)	Human 20–30 years	5	Nasal biopsy	(1) Simple dissection and mincing. (2) Tissue washing with antibiotics. (3) Direct plating of the tissue. (4) Basic medium plating. (5) Layer.
Simorgh et al. (2019)	Wistar Rats 8 weeks old	N/A	Nasal biopsy	(1) Complex dissection and mincing. (2) Tissue direct plating. (3) Basic medium plating. (4) Replating of cells in the following passages.
Kim et al. (2019)	BALB/c Mice 4 weeks old	5	Nasal biopsy	(1) Simple dissection and mincing (pull 5 mice). (2) Tissue washing with antibiotics. (3) Enzymatic and mechanical dissociation. (4) Complex medium plating. (5) Spheres mechanical dissociation.
Esaki et al. (2019)	Mice Neonatal	N/A	Nasal biopsy	(1) Simple dissection and mincing (pull 5 mice). (2) Tissue washing with antibiotics. (3) Dissociation by 70 μm cell strainer. (4) Mechanical dissociation. (5) Complex medium plating. (6) Fresh medium was added to the cultures every third day. (7) Spheres.
Salehi et al. (2019)	Human	N/A	Nasal biopsy	(1) Simple dissection and mincing (pull 5 mice). (2) Tissue washing with antibiotics. (3) Direct tissue plating. (4) Basic medium plating. (6) Change the medium every 3 days. (7) Layer.
Alizadeh et al. (2019b)	Human	N/A	Nasal biopsy	(1) Complex dissection and mincing, enzymatic treatment. (2) Direct tissue plating. (3) Basic medium plating. (4) Layer.
Bagher et al. (2019)	Human	2	Nasal biopsy	(1) Simple dissection and mincing. (2) Tissue washing with antibiotics. (3) Direct tissue plating. (4) Basic medium plating. (5) Dissociation replating. (6) Change the medium every 3 days. (7) Layer.
Alizadeh et al. (2019a)	Human 20–30 years	5	Nasal biopsy	(1) Simple dissection and mincing. (2) Tissue washing with antibiotics. (3) Direct tissue plating. (4) Basic medium plating. (5) Dissociation replating. (6) Change the medium every 3 days. (7) Layer.
Solís-Chagoyán et al. (2019)	Human	54 years old healthy woman	Nasal brush	(1) Exfoliation. (2) Cells washing with antibiotics. (3) Basic medium plating. (4) Dissociation replating. (5) Change the medium every 3 days. (6) Layer.
Pérez-Luz et al. (2020)	Human	7 total 3 healthy donors 4 FRDA patients	Nasal biopsy	(1) Simple dissection and mincing. (2) Tissue washing with antibiotics. (3) Direct tissue and cells plating. (4) Complex medium plating. (5) After 3 days growing remove non adherent cells. (6) Dissociation replating. (7) Change the medium every 3 days. (8) Layer.
Alvites et al. (2020)	Sprague Dawley Rat 8–9 weeks	10	Nasal biopsy.	(1) Simple dissection and mincing. (2) Direct tissue plating. (3) Basic medium plating. (4) Passaged by trypsin. (5) Layer.
Jafari et al. (2020)	Human	10	Nasal biopsy	(1) Simple dissection and mincing. (2) Enzymatic dissociation. (3) Basic medium plating. (4) After 72 h, non-adherent cells removed with PBS. (5) Medium changed every 3 days. (6) Layer.
Tian et al. (2020)	C57BL/6 Mice 6–8 weeks	N/A	Nasal biopsy	(1) Simple dissection and mincing. (2) Direct tissue plating for 7 days. (3) Basic medium plating. (4) Passaged by trypsin. (5) Layer.
Liu et al. (2020)	Human 20–40 years	4	Nasal biopsy	(1) Simple dissection and mincing. (2) Tissue washing with antibiotics. (3) Direct tissue plating. (4) Basic medium plating. (5) Passaged by trypsin. (6) Layer.
Voronova et al. (2020)	Human	40	Nasal biopsy	(1) Complex dissection and mincing. (2) Direct tissue plating. (3) Basic medium plating. (4) Passaged by trypsin. (5) Basic medium plating added with FGF2 and EGF. (6) Layer.
Farhadi et al. (2021)	Human	N/A	Nasal biopsy	(1) Simple dissection and mincing. (2) Tissue washing with antibiotics. (3) Direct tissue plating. (4) Basic medium plating. (5) Remove non adherent cells. (6) Passaged by trypsin. (7) Layer.
Hamidabadi et al. (2021)	Human	N/A	Nasal biopsy	(1) Simple dissection and mincing. (2) Tissue washing with antibiotics. (3) Direct tissue plating. (4) Basic medium plating. (5) Remove non adherent cells. (6) Passaged by trypsin. (7) Layer.
Zelenova et al. (2021)	Human 20–45 years	11	Nasal biopsy	(1) Complex dissection and mincing. (2) Direct tissue plating. (3) Basic medium plating. (4) Passaged by trypsin. (5) Complex medium plating. (6) Spheres.
He et al. (2021)	Human	3	Nasal biopsy	(1) Simple dissection and mincing. (2) Basic medium plating. (3) Layer.
Ren et al. (2021)	Human and Mice (3 months old)	N/A	Humans: Nasal Biopsy Mice: genetically targeted mice (GFP) olfactory epithelium was purchased	(1) Simple dissection and mincing (pull 5 mice). (2) Tissue washing with antibiotics. (3) Enzymatic dissociation. (4) Mechanical dissociation by 70 μm cell strainer. (5) Mechanical dissociation. (6) Complex medium plating. Cells were cultivated in DMEM/F12 medium supplemented with R-spondin-1, Noggin, Wnt3a, Y27632, GlutaMAX™ Supplement, HEPES, and Matrigel. (7) Colonies dissociated by trypsin.
Mollichella et al. (2022)	Cat Median 6 ± 4.6 years	4	Nasal biopsy	(1) Simple dissection and mincing. (2) Tissue washing with antibiotics. (3) Complex medium. (4) Change the medium every 2 days. (5) Layer.
Rövekamp et al. (2022)	Human	5	Nasal biopsy	(1) Simple dissection and mincing. (2) Tissue washing with antibiotics. (3) Enzymatic dissociation. (4) Complex medium: (5) Change the medium every 2 days. (6) Spheres.
Wang et al. (2022)	mice	N/A	Nasal biopsy	(1) Basic dissection and mincing. (2) Tissue washing with antibiotics. (3) Direct tissue plating. (4) Basic medium plating. (5) Passaged by trypsin. (6) Complex medium plating. (7) Layer.
Rantanen et al. (2022)	Human	6 affeccted by AD and 6 CTR	Nasal biopsy	(1) Basic dissection and mincing. (2) Tissue washing with antibiotics. (3) Complex medium plating. (4) Passaged by trypsin. (5) Complex medium plating. (6) Spheres.
Entezari et al. (2022)	Human	N/A	Nasal biopsy	(1) Complex dissection and mincing. (2) Tissue washing with antibiotics. (3) Enzymatic dissociation. (4) Basic medium plating. (5) Passaged by trypsin. (6) Complex medium plating. (7) Spheres.
Unzueta-Larrinaga et al. (2023)	Human	8	Nasal brush	(1) Exfoliation. (2) Tissue washing with antibiotics. (3) Complex medium plating. (4) Passaged by trypsin. (5) Complex medium plating. (6) Spheres.

This table summarize of the selected studies, with the description of the Type of mammal, their age, the sample population, the harvesting technique, and the culturing technique. Ref# = Reference number. Nasal Brushing: Exfoliation: Using a specific brush with a length of 2.4 cm and a diameter of 3 mm (these can slightly vary from protocol to protocol), cells were exfoliated from the anterior area of the medial lateral tubule and the lateral wall of the nasal cavity and septum by making circular motions. Nasal biopsy: Simple dissection and mincing: Mouse tissue fragments from olfactory turbinate blocks and septums were dissected. Complex dissection and mincing: Mouse tissue fragments from olfactory turbinate blocks and septums were dissected, lamina propria was separated under a dissection microscope. Tissue washing with antibiotics: PBS or DMEM/F-12 (added with 10% FBS or FCS) containing 100 g/mL penicillin and 100 IU/mL streptomycin. Mechanical dissociation: Culture was dissociated mechanically up to 40 times through a micropipette tip. Enzymatic dissociation: Olfactory mucosa was dissociated by incubating in collagenase or Dispase II, DNAse (alternative) for 30 or more min at 37 °C. Following, we have the enzyme inactivation. Cell cultivation: Basic medium: DMEM/F-12 (added with 10% FBS or FCS). Complex medium: DMEM/F12 supplemented with epidermal growth factor (EGF, 20 ng/mL) and fibroblast growth factor (FGF2, 20 ng/mL). NSM contained DMEM/F12 (1:1), supplemented with 0.105% NaHCO3, L-glutamine (2 mM, Invitrogen), penicillin (5,000 IU/mL), streptomycin (5 mg/mL, Invitrogen), glucose (5 mM), insulin (25 mg/mL, Sigma), apotransferrin (100 mg/mL), putrescine (60 mM), progesterone (20 nM), and sodium selenite (30 nM) at 37 °C. The concentration of the different components can slightly vary. CO2 can be 5 or 7%. Medium change or feeding can slightly vary from paper to paper. Cells: Layers, Neurospheres, The amount of cells for replating can vary. N/A: Not available.

Articles were selected based on title and abstract and, when needed, on inspection of the full text to assess relevance. Records retrieved through references of these articles were screened for additional studies not previously identified without obtaining any further researches to be included in the systematic review as olfactory stem cell isolation is a fairly novel investigation area.

We included any study that has an in-depth assessment focused on the isolation of NSCs from the olfactory mucosa of mammals, with sampling taken either by brushing or biopsy. The inclusion criteria considered were a sample taken from a mammalian olfactory mucosa, a well-described process of the harvesting of the sample to be analyzed, obtained either by brushing or biopsy, as well as the post-harvesting manipulation like mechanical dissociation, detailed explanation on the culture medium used and addition of any other substances, and finally laboratory-confirmed isolation of stem cells with identification of the known markers of the olfactory stem cells. We excluded from our study all meta-analyses, systematic and narrative reviews, studies showing isolation of olfactory mucosa cells other than NSCs or isolation of NSCs from samples harvested from other areas of the nose like respiratory epithelium, olfactory bulb, or vomeronasal organ, articles on case/control studies solely showcasing the differences in the olfactory mucosa stem cells in healthy versus different pathologies, absence of explanation of the method used to evaluate the features of the culture-derived cells, and articles solely focusing on the therapeutic application of the stem cells obtained from the olfactory epithelium without mentioning the process of their isolation. No minimum study population was required.

**Figure 1:**
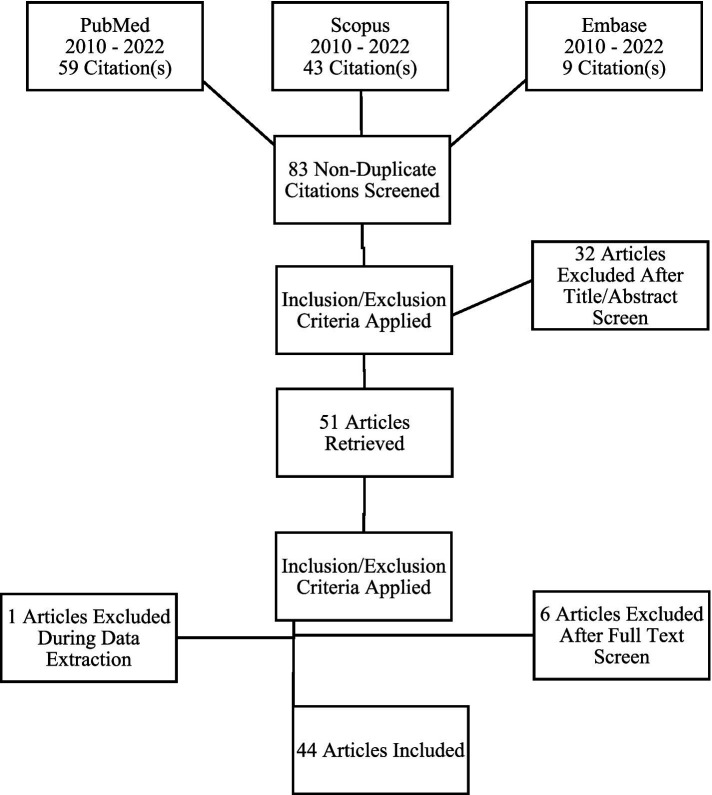
PRISMA chart depicting the search strategy for the systematic review.

A total of 44 articles fulfilled the inclusion and exclusion criteria and were included in this review (Figure 1).

The research process was conducted by two different authors (C. P. (MD), P. K. (MD)). Abstracts and full texts were reviewed thoroughly. To maximize the rate of inclusivity, in the early, abstract stage, of the review we included all studies deemed eligible by at least one rater. Then, at the full-text review stage, the two authors closed the final selection of the studies, by discussing disagreements and finding a final consensus.

PICOS criteria.

The PICOS criteria for the present review are as follows:

P: Mammals.
I: Biopsy and/or Brushing of the nasal olfactory epithelium.
C: No comparison available.
O: Isolation of NSCs.
S: Original studies of any kind and clinical setting (except reviews and meta-analysis).

### Data extraction and quality assessment

Data extraction process: Two reviewers (C. P. and P. K.) independently extracted data from all eligible full texts using a standardized data-extraction form (Microsoft Excel). Extracted items included: species, donor characteristics/sample size, sampling technique (biopsy vs. brushing, anatomical site), pre-processing (washing, mechanical/enzymatic dissociation), culture conditions (basic vs. complex medium, serum vs. serum-free, adherent vs. sphere culture), passage/expansion details, markers used for characterization, and outcomes related to proliferation and lineage differentiation. Any discrepancies between reviewers were resolved first by discussion. Final entries were cross-checked prior to synthesis.

Risk of bias was assessed for all included studies using the SYRCLE’s Risk of Bias (RoB) tool for animal studies, following published guidance with slight adaptations to account for the frequent use of *in vitro* and culture-based experimental designs. Each article was independently evaluated by two reviewers across key domains including sequence generation/randomization, allocation concealment (when applicable), blinding during sample handling and outcome assessment, completeness of outcome data, selective reporting, and other sources of bias (e.g., standardization of culture conditions and appropriateness of controls). Any disagreements were resolved by discussion, with involvement of a third reviewer when needed. The risk-of-bias results were summarized in Tables 2, 3 and used to contextualize the interpretation of methodological heterogeneity and overall strength of evidence.

**Table 2:** Animals.

Domains/ Paper#	1	2	3	4	5	6	7	8	9	10	11	12
Krolewski et al. (2011)	+	−	++	−	++	++	++	++	++	++	++	++
Toft et al. (2012)	+	−	++	−	++	++	−	++	++	−	+	+
Stamegna et al. (2014)	+	−	++	−	++	+	−	+	+	+	+	+
Thakur et al. (2014)	+	−	++	−	++	++	−	++	++	−	+	++
Altunbaş et al. (2016)	+	−	+	−	+	+	−	+	−	−	+	+
Ercolin et al. (2016)	+	−	++	−	++	++	−	+	++	−	+	++
Franco et al. (2017)	++	−	++	++	−	++	+	++	++	++	++	++
Muniswami et al. (2017)	++	−	++	−	++	++	−	++	++	++	++	++
Veron et al. (2018)	++	−	+	−	++	++	−	+	+	−	+	++
Li et al. (2018)	+	−	++	−	++	++	−	++	++	++	++	++
Simorgh et al. (2019)	++	−	++	++	++	++	++	++	++	++	++	++
Kim et al. (2019)	+	−	++	−	++	++	−	++	++	+	++	++
Esaki et al. (2019)	++	−	++	−	++	++	−	+	+	++	+	++
Alvites et al. (2020)	+	−	++	−	++	++	−	−	+	++	++	++
Tian et al. (2020)	+	−	+	−	++	++	−	++	++	++	++	++
Ren et al. (2021)	+	−	++	−	++	+	−	++	++	++	++	++
Mollichella et al. (2022)	+	−	++	−	++	++	−	++	++	−	+	+
Wang et al. (2022)	+	−	+	−	++	++	−	++	++	++	++	++

++ very low risk; + low risk, − unclear risk, high risk.

**Table 3:** Human.

Domains/ paper#	1	2	3	4	5	6	7	8	9	10	11	12
Benítez-King et al. (2011)	++	−	++	−	++	++	−	++	++	++	++	++
Lindsay et al. (2013)	++	−	++	−	++	++	−	++	++	++	++	++
Batioglu-Karaaltin et al. (2016)	+	−	++	++	++	++	+	++	++	++	+	++
Ge et al. (2016)	+	−	++	−	++	++	+	++	++	+	++	++
Tanos et al. (2017)	+	−	++	−	++	++	+	++	++	++	++	++
Franco et al. (2017)	++	−	++	++	−	++	+	++	++	++	++	++
Jiménez-Vaca et al. (2018)	++	−	++	−	++	++	+	++	++	−	+	+
Bagher et al. (2018)	+	−	+	−	++	++	+	++	+	++	+	++
Salehi et al. (2019)	++	−	++	−	++	++	−	++	++	++	++	++
Alizadeh et al. (2019a)	++	−	++	−	++	++	+	++	++	++	++	++
Bagher et al. (2019)	+	−	+	−	++	++	+	++	++	++	++	++
Alizadeh et al. (2019a)	+	−	+	−	++	++	+	++	++	++	++	++
Solís-Chagoyán et al. (2019)	−	−	+	−	++	++	+	++	+	++	++	++
Pérez-Luz et al. (2020)	++	−	++	−	++	++	+	++	++	++	++	++
Jafari et al. (2020)	++	−	+	−	++	++	+	+	++	++	++	++
Liu et al. (2020)	+	−	++	++	++	++	+	++	+	++	++	++
Voronova et al. (2020)	++	−	+	−	++	++	+	++	++	++	++	++
Farhadi et al. (2021)	++	−	++	−	++	++	+	++	++	++	++	++
Hamidabadi et al. (2021)	++	−	++	−	++	++	−	++	++	++	++	++
Zelenova et al., 2021	++	−	+	−	++	++	−	++	++	+	++	++
He et al. (2021)	+	-.	+	−	++	++	−	++	++	++	++	++
Rövekamp et al. (2022)	+	−	+	−	++	++	−	++	++	−	++	++
Rantanen et al. (2022)	++	−	++	−	++	++	−	++	++	++	++	++
Entezari et al., 2022	+	−	+	−	+	+	−	++	+	++	++	++
Unzueta-Larrinaga et al., 2023	+	−	++	−	+	+	−	++	++	++	++	++

++ very low risk; + low risk, − unclear risk, high risk.

For each included article we recorded the mammal used, the sample population, the technique used for harvesting the sample with details of the procedure and location within the nasal cavity, country – and city if available – in which the study was performed, the type of study, research data whenever available, the culture medium used, details on the preparation of the culture, the markers targeted in the study to evaluate the characteristics of the culture-derived cells, and finally the methods used for the evaluation of the culture.

Quantitative synthesis of included studies: Among the 44 included studies, 27 (61.4%) were conducted in humans and 17 (38.6%) in other mammals. Sampling relied predominantly on biopsy (39/44, 88.6%), whereas nasal brushing was used in only 5/44 studies (11.4%). Regarding culture conditions, basic and complex media were equally represented (22/44 each, 50.0%), with a slightly higher use of adherent monolayer cultures (23/44, 52.3%) compared with sphere-based cultures (21/44, 47.7%). Serum-free conditions were reported in 16/44 studies (36.4%), reflecting a gradual trend toward clinically compliant protocols.

## Results and analysis

From the 83 non-duplicate research items initially identified, 44 original research articles (Figure 1; Table 1) were finally selected, with 10 different types of mammals used as specimens. The mammals used were humans, rats, mice, dogs, cats, rabbits, sheep, horse, gray mouse lemur, and macaque, with the majority of the studies focusing on humans (27 out of 44). Most of the selected studies were carried out in Asian and European countries.

Seven out of 44 (15.1%) of the outcomes reported in the chosen studies showed that these cells had excellent applications for nervous system transplantation or treatment (13 out of 44, 29,5%).

In fact, only 11.4% (5 out of 44) of the cells generated by the various investigations characterized a non-neural lineage, whereas the majority (9 out of 44, 20.5%) exhibited the hallmark of a neural lineage. An *in vivo* neural differentiation capability (11.4%) was discovered in five publications; the majority of it (9.1%) was focused on dopaminergic neurons (or the cells were employed for Parkinson’s treatments in animal models).

### Risk of bias in included studies

We conducted a risk-of-bias assessment using SYRCLE’s RoB tool following the criterias indicated below following (Hooijmans et al., 2014; Hameete et al., 2025), with slight modifications:

1) Randomization: Were groups or samples randomized? (For instance, use random number generators).
2)Sample Size: Was the sample size (power calculation, for example) justified?
3) Controls: Are suitable positive and negative controls present?
4) Blinding: Were samples handled and prepared by researchers without regard to the group?
5) Standardization: Manufacturer specifications for materials, consistent operator, and circumstances (media, temperature)?
6) Sample Integrity: Reducing cell deterioration or death prior to or during the experiment?
7) Blinding: Were readers and result assessors blinded to treatment groups?
8) Objective Measures: Do you employ quantitative, objective, and established methods?
9) Consistency: Do all samples use the same measuring methods?
10) Statistical Methods: Is the right analysis applied (for non-normal data, for example)?
11) Completeness: Were all samples and data examined, with no inexplicable exclusions or dropouts?
12) Transparency: Explicit reporting of procedures, findings (even unfavorable ones), and restrictions (derived from CRIS/CONSORT).

For every study included in this systematic review, the risk of bias was evaluated. A third reviewer was consulted if needed, and any differences between the two writers’ independent evaluations were settled by discussion.

Every one of the included studies underwent a risk of bias evaluation. A description of this assessment’s results is presented in Tables 2, 3. As can be observed, almost all the studies had extremely little chance of selection bias. This is partly because there is a lot of research that employs cell cultures, which are often cultivated and used as a single, homogeneous cell suspension and are hence immune to selection bias, and it was avoided the danger of selection bias and biological duplication. The risk of bias was also typically very low for research employing original material. However, in a few instances, it was impossible to ascertain. The only item where the bias was uncertain was the sample size that was not determined in any paper.

## Discussion

Our systematic review strongly suggests that cells with the potential to originate neural cells and their differentiated progeny *in vitro* are present in the olfactory mucosa (OM). This is indicative of the presence of neurogenetic activity in this region. Furthermore, these cells showed the characteristics of progenitor stem cells *in vitro,* as they express neural progenitor cell markers and the capacity to differentiate *in vitro*, although more functional electrophysiological studies are needed (Durante et al., 2020; Jaloux et al., 2022; NIH, 2024; Carrillo González et al., 2025; Park and Song, 2024; Teixeira et al., 2017).

OM-MSCs exhibit a unique combination of neural, glial, and mesenchymal markers, which underscores their pluripotent nature and suitability for applications in regenerative medicine. Studies consistently report the expression of markers such as Nestin, Sox2, GFAP, and S100, alongside traditional mesenchymal markers like CD44 and CD105 (Table 4). These findings indicate that OM-MSCs indeed have neurogenic properties, allowing them to be particularly suitable for neural repair (Durante et al., 2020; NIH, 2024; Jaloux et al., 2022; Park and Song, 2024; Abdelbaset et al., 2024; King and Perrin, 2014). The presence of these markers also indicates their capacity for multilineage differentiation, which is critical for developing therapeutic strategies.

**Table 4:** Summary of targets and the methods used to analyze them in culture-derived cells.

Study and year	Markers targeted	Evaluation of tissue and culture-derived cells
Benítez-King et al. (2011)	TUJ1, nestin, GFAP, vimentin, and OMP	Immunofluorescence. Western blot. Differentiation assay. Electrophysiological characterization.
Krolewski et al. (2011)	Sox2, Sox9, E-cadherin, CD54, PGP9.5, CK18, CK14, Ki67, NCAM, *α*—neuron specific tubulin, PCNA, Vimentin, Thy 1.2, PECAM.	Immunocytochemistry. Immunohistochemistry. FACS. Transplantation.
Toft et al. (2012)	Nestin, p75^NTR^, CD90, Fibronectin, CD105, STRO-1, SMA, cytokeratin.	Immunocytochemistry. Transplantation.
Lindsay et al. (2013)	CD54, CD73, CD90, CD105, CD106, CD166, CD271, STRO-1, Nestin, NG2, GFAP, SMA, citokeratin 5/6, Tuj-1.	Immunohistochemistry. Clonal efficiency. 5-Bromo-2-Deoxyuridine treatment. Osteogenic and Adipogenic Differentiation. Myelination and Quantification of Myelination.
Stamegna et al. (2014)	Nestin, S100A4.	Immunostaining. Olfactory associative discrimination task.
Thakur et al. (2014)	GBC, NCAM, Nestin, Sox-1, Sox-2, CD29, CD54, CD73.	FACS. Immunohistochemstry. Electrophysiological characterization.
Ohnishi et al. (2015)	AN2 biotin conjugated, A2B5 biotin conjugated, TUJ1, GAD67, GFAP, MAP2, NF, RIP, Olig2, O4, Tyrosine hydroxylase.	Immunocytochemistry. FACS.
Altunbaş et al. (2016)	Nestin, *β*III-tubulin, GFAP, NCAM, NANOG, OCT4, SOX2, GAPDH.	Total RNA Isolation. Real time PCR.
Ercolin et al. (2016)	GFAP, CD34, CD45, CD73, CD79, CD90, CD105.	Growth curve. Cryopreservation. Colony forming unit like fibroblasts (CFU-f). Differentiation protocols (Osteogenic, Adipogenic, Neuronal). Immunofluorescence. FACS.
Batioglu-Karaaltin et al. (2016)	CD14, CD117, CD11b, CD13, CD44, CD90, CD116, CD146, CD73, HLA-ABC-PE, CD29, CD105, HLA-DR-FITC.	Immunostaining. FACS. Differentiation into adipocytes. Electrophysiological characterization.
Ge et al. (2016)	Nestin, STRO-1, MAP-2, CD34, CD45, CD73, CD90, and CD105.	Immunofluorescence. Flow cytometry. Osteogenic, Adipogenic, and Neurogenic differentiation. Protein extraction. SDS-PAGE and Western blotting. Tryptic digestion and MS/MS analysis.
Tanos et al. (2017)	CK14, CK5, CK8, Epcam, FN, Ki67, Nestin, NSE, p63, S100, S100*β*, Synaptophysin, SMA, TUJ1, Vimentin. Vinculin.	Immunohistochemistry. FACS. Western blot. Immunocytochemistry. Real Time PCR. Affymetrix GeneChip hybridization. Stem cell purification.
Franco et al. (2017)	MAP1B, MAP2, Nestin, *β*III-tubulin, Neuron specific enolase (NSE), Spx2, Klf4, Nanog, Lin28, Dnmt1.	Cell proliferation and Viability assay. Sphere formation. Neuronal differentiation. Real Time PCR. Neurite outgrowth analysis.
Lu et al. (2017)	CD34-ECD, CD45-PC7, CD73, CD90, CD105.	Flow cytometry. Cell-growth curve measurement. Cell cycle analysis. *In vitro* induced mesenchymal differentiation. Immunofcitochemistry. Western Blot.
Muniswami et al. (2017)	GBC-3, Nestin, SOX2, CD54, CD90, CD73, CD29, CD105, CD45, CD31, TUJ1, MAP2, NeuN, NF.	FACS. Immunohistochemistry. Immunocytochemistry. Transplantation.
Veron et al. (2018)	Nestin, Tenomodulin, Scleraxis, GFAP, MAP2, CD34, CD44, CD73.	Immunocytochemistry. FACS. Clonal efficiency assay. *In vitro* proliferation assay. In vitro mesodermal differentiation assay.
Li et al. (2018)	Ascl1, βIII-tubulin, OMP, Olfactory neuron specific-G protein, ADCY3, BrdU.	Real Time PCR. Immunocytochemistry. Western blot.
Jiménez-Vaca et al. (2018)	Nestin, Sox2, Musashi, TUJ1, Cytokeratin, GFAP, OMP.	Immunocytochemistry. Real Time PCR. Clonal analysis. Differentiation analysis.
Bagher et al. (2018)	Vimentin, Nestin, CD73, CD90, CD105, CD34, CD45, ChAT, Hb-9, Islet-1, GAPDH.	Real Time PCR. Immunocytochemistry. Adipogenic and Osteogenic differentiation. FACS. Motor-neuron differentiation.
Simorgh et al. (2019)	CD90, CD44, CD34, CD45, Nestin, Vimentin.	Flow cytometry. Immunocytochemistry. Immunohistochemistry. Real Time PCR. Transplantation.
Kim et al. (2019)	LGR5, Musashi, SOX2, NCAM, OMP, TUJ1, GFAP, O4, NeuroD1, phosphor-p38, IL5R, TNFαR, β-actin.	FACS. Immunocytochemistry. Cell growth and viability analysis. Western Blot.
Esaki et al. (2019)	Nestin, Musashi-1, Sox2, Nanog, Oct3/4, OMP, GAPDH, TUJ1, GalC, GFAP, OMP.	Real time PCR. Immunocytochemistry. Electrophysiological characterization. Transplantation.
Salehi et al. (2019)	CD105, CD90, CD73, CD34, CD45, Nestin, Vimentin.	FACS. Immunocytochemistry. Osteogenic and Adipogenic differentiation.
Alizadeh et al. (2019b)	CD105, CD90, CD73, CD45, CD34, Nestin, Vimentin, Map2, Tyrosine hydroxylase (TH), Dopamine transport (DAT), Pitx3, CD34, EN1, EN2, NURR1, WNT1, LMX1A, GAPDH.	FACS. Immunocytochemistry. Immunocytochemistry. Adipogenic and Osteogenic differentiation. Dopaminergic neuron differentiation. Real-time PCR.
Bagher et al. (2019)	CD105, CD90, CD73. CD45, CD34, Nestin, Islet-1, Chat, HB9, GAPDH, Choline acetyltransferase	Facs. Adipogenic and Osteogenic differentiation. Differentiation into motor-neurons. Real-time PCR. Immunofluorescence.
Alizadeh et al. (2019a)	CD105, CD90, CD73, CD34, CD45, Nestin, MAP2, TH, DAT, PITX3, NURR1, WNT1, PAX3, EN1, EN2, GAPDH.	Cell proliferation assay. Adipogenic and Osteogenic differentiation. FACS. Real-time PCR, Immunohyistochemistry, HPLC.
Solís-Chagoyán et al. (2019)	Nestin, vimentin, olfactory marker protein (OMP), neuronal enolase, BrdU, P2X1–5, P2X7, P2X3, P2X4, P2Y2, P2Y4, P2Y6, P2Y11.	Proteome Profiler™ Human Pluripotent Stem Cell Array Kit. Immunocytochemistry. ELISA. Western Blot.
Pérez-Luz et al. (2020)	Stro-1, Nestin, NG2, CD133. CD49D, Nanog, Klf4, Sox9, Sox2, Pax3, TrkB, PCTH1, phosphorylated MAP1B, Tuj-1, SMI31, Reactive Oxygen Species	Immunocytochemistry. Real-time PCR. Cell viability assay. Differentiation into Neural cells.
Alvites et al. (2020)	Cytokeratin, Vimentin, CD31, Synaptophysin, c-Kit, GFAP, NANOG, Oct4, Sox2, CD105, CD90, CD73, CD44, CD45, CD34, BSP, Runx2, NGF and GDNF, Tenomodulin, Desmin	Growth curve and Cell viability. Population Doubling Time (PDT). Colony forming unit assay. Differentiation – Adipogenic, Chondrogenic. Osteogenic and Neurogenic). Real-time PCR. Cytogenetic analysis. Imunohistochemistry.
Jafari et al. (2020)	CD14, CD31, CD34, CD45, CD44, CD73, CD90, CD105, HLA-DR, TLR3, TLR4, Nestin, Vimentin, N-cadherin, GAPDH	Immunocytochemistry. Real-time PCR. Adipogenic and Osteogenic differentiation. Population doubling time.
Tian et al. (2020)	CD29, CD90, CD44, CD34, CD45, CD11b, CD4, IFN-*γ*, IL-17, CD25, Foxp3, Nestin, Vimentin	Adipogenic and Osteogenic differentiation. FACS. Nanoparticle Tracking Analysis. Western blot. Electron microscopy.
Liu et al. (2020)	CD105, CD90, CD73, CD44, CD146, CD133, CD34, CD45, SOD, GSH-PX, MDA, LPO, CAT, UBIAD1	Facs. Oxidative stress level. Tunel assay. Mitochondrial function. Western blot. Real Time PCR.
Voronova et al. (2020)		Transplantation Behavioral study
Farhadi et al. (2021)	CD105, CD90, CD34, CD45, Nestin, Vimentin, VEGF, GDNF, DAT, Nurr1, TH	FACS. Immunocytochemistry. Osteogenic and Adipogenic differentiation. Western blot analysis. Transplantation. Behavioral study. Electromyography.
Hamidabadi et al. (2021)	CD105, CD90, CD73, CD45, CD34, Nestin, Vimentin, MAP2, NF-200 kDa, ChAT, Oct4, Sox2, NF-M, MF-H, Islet1, Olig2, HB9, GAPDH.	FACS. Immunocytochemistry. Osteogenic and Adipogenic differentiation. Motor-neuron differentiation. Western blot analysis. Transplantation. Real Time PCR.
Zelenova et al. (2021)	Nestin, GFAP, TAGLN, Actin, α-Smooth muscle.	Immunocytochemistry. RNA-sequencing and Expression analysis.
He et al. (2021)	CD44, CD73, CD90, CD105, CD133, CD146, CD34, CD45.	FACS. Real time PCR. qRT-PCR. Profiling of DElncRNAs and DEmRNAs. Cell viability. Cell cycle measurement. Apoptosis measurement. Western blot assay.
Ren et al. (2021)	OMP, Tuj-1, Sus4, GFP, Mash1, Sox-2, Ki67, Gαolf, mOREG/MOR174-9, PGP9.5, human Lgr5, human Krt5, mouse GAPDH.	FACS. Colony culture. Neuronal differentiation. Real time PCR. Western blot. RNA sequencing. Calcium imaging.
Mollichella et al. (2022)	Nestin, GFAP, MAP2, Tenomodulin, Scleraxis.	Immunocytochemistry. Neural differentiation assay. Clonal efficiency assay, In vitro proliferation assay (PDT). In vitro mesodermal differentiation assay. (osteogenic, chondrogenic and tenogenic).
Rövekamp et al. (2022)	Nestin, CD90, CD44, CD105, Ki-67, Doublecortin, GFAP, Tuj1	FACS. Immunofluorescence. Neuronal differentiation. Immuhistochemistry
Wang et al. (2022)	Nestin, Vimentin, and S100.	Western Blotting. Immunoflourescence. Immunohistochemistry.
Rantanen et al. (2022)	Nestin, PAX6, TUJ1, MAP2, DCX, AQP4 and S100B.	Immunocytochemistry. Real time PCR.
Entezari et al. (2022)	CD105, CD90, CD45 CD34, nestin and vimentin, SOX10, p75, S100, GFAP, MBP, BDNF, NGF.	Immunocytochemistry. FACS. Real time PCR. ELISA.
Unzueta-Larrinaga et al. (2023)	Nestin, Sox2, Musashi-1, PSA-NCAM, TUJ1, Neuronal-Nuclei (NeuN), MAP2, MAP1B, GFAP, EpCAM, PGE2, IL6.	Immunocytochemistry. FACS. Real time PCR. ELISA.

This table summarizes the molecular markers and analytical methods used across each study which investigates olfactory mucosa derived stem cells. It shows that most authors evaluated both neural progenitor markers (e.g., Nestin, Sox2, Musashi, TUJ1, MAP2, and NeuN) and glial markers (GFAP, S100, vimentin), often alongside MSCs surface antigens (CD73, CD90, CD105, Stro-1). Several studies also included pluripotency-associated genes (Oct4, Nanog, Klf4), lineage-specific markers such as dopaminergic (TH, DAT, Pitx3, Nurr1) or motor neuron–related proteins (Islet-1, ChAT, HB9), and disease-relevant factors linked to oxidative stress, inflammation, or mitochondrial function. Immunocytochemistry was the most frequent evaluation techniques, complemented by flow cytometry (FACS), Western blotting, and real-time PCR/qRT-PCR. Ref# = reference number.

The neurogenic potential of OM-MSCs has been further demonstrated through their ability to secrete neurotrophic factors such as Brain-Derived Neurotrophic Factor [BDNF, Nerve Growth Factor (NGF), and Glial-Derived Neurotrophic Factor (GDNF)] (Carrillo González et al., 2025). These paracrine effects contribute to the survival and regeneration of damaged neural tissues, positioning OM-MSCs as both direct contributors to tissue repair and enhancers of endogenous repair mechanisms.

**Figure 2:**
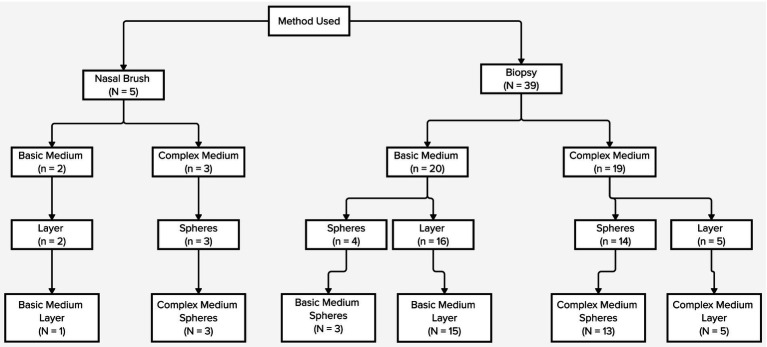
Flow chart summarizing the correlation between sampling method (biopsy vs. brushing), culture medium (basic vs. complex), and resulting growth pattern (adherent monolayer vs. sphere formation).

Despite slight differences in harvesting and culturing methodologies, most studies reported consistent results in isolating these cells. The predominant method—biopsy—was employed in 88.6% of studies (39 out of 44), supporting the idea that deeper sampling yields higher numbers of stem cells (Table 5; Supplementary Table S1). This is likely because deeper tissue layers contain a greater density of these cells. Interestingly, the research allows the production of spheres (in many cases, neurospheres) or layers. This is most likely due to the method of sampling but also from the type of medium used. Indeed, we found that the complex medium allows the formation of spheres, whereas the basic medium allows with more chances the formation of a monolayer. See Table 6 and Figure 2.

**Table 5:** Results of the analysis of the culture derived cells.

Study and year	Results of the analysis
Benítez-King et al. (2011)	Neural precursor bank; neuronal developmental cytoskeletal abnormalities.
Krolewski et al. (2011)	Conditioned media; media effects on ONSs; cell fate; transplantation.
Toft et al. (2012)	ONSs; transplantation; high astrocytic hypertrophy.
Lindsay et al. (2013)	LP-MSCs; oligodendrocyte myelination; *in vitro*.
Stamegna et al. (2014)	Rat OM-MSCs; transplantation.
Thakur et al. (2014)	Globose Basal cells (GBCs); neural progenitor cells markers; not excitable.
Ohnishi et al. (2015)	ONSs; neural progenitors; differentiation; transplantation.
Altunbaş et al. (2016)	OM-MSCs; canine; bone marrow; adipose tissue.
Ercolin et al. (2016)	OM-MSCs; mesenchymal cells; CD34; CD73; CD90; prolonged in vitro culturing life.
Batioglu-Karaaltin et al. (2016)	hOM-MSCs; transplantation in rat; higher whisker-movement scores.
Ge et al. (2016)	hOM-MSCs; secretome analysis; 274 secreted proteins; neurotrophy; angiogenesis; cell proliferation; differentiation; apoptosis; inflammation; transplantation.
Tanos et al. (2017)	hOM-MSCs; stem cells production; characterization.
Franco et al. (2017)	hOM-MSCs; pluripotency.
Lu et al. (2017)	hOM-MSCs; mesenchymal marchers; rhodopsin photoreceptor-specific marker.
Muniswami et al. (2017)	GBCs; NSC markers; mesenchymal stem cell markers, neuronal differentiation.
Veron et al. (2018)	OM explants; CD44; CD73; mesodermal lineages.
Li et al. (2018)	OM-MSCs; neurospheres; chitosan films; immature and adult olfactory receptor; neurons in olfactory ONSs.
Jiménez-Vaca et al. (2018)	OM exfoliation; NSCs.
Bagher et al. (2018)	OM-MSCs; stem cells; motor neurons differentiation.
Simorgh et al. (2019)	PD model; OM-MSCs; transplantation; Parkinson’s improvements; (PAX2, PAX5, PITX3, dopamine transporter, tyrosine hydroxylase increase).
Kim et al. (2019)	ONSs; neural stem cell markers (Tuji1, GFAP, and O4); allergic rhinitis model; neurospheres number reduction.
Esaki et al. (2019)	OM-MSCs; FNP; transplantation; mouse model improvements.
Salehi et al. (2019)	OM-MSCs; alginate/chitosan; rat sciatic nerve model; enhanced regeneration.
Alizadeh et al. (2019b)	OM-MSCs; dopamine neuron markers expression; dopamine release.
Bagher et al. (2019)	OM-MSCs; hydrogels; motor neurons.
Alizadeh et al. (2019a)	OM-MSCs accessibility; Wharton’s jelly area (WJ)-MSCs; comparison; OE-MSCs higher proliferation, differentiation; dopaminergic neurons.
Solís-Chagoyán et al. (2019)	hOM-MSCs purinergic (P2) receptors expression; exocytosis and Ca^2+^ signaling.
Pérez-Luz et al. (2020)	hOM-MSCs; Friedreich’s ataxia patients; decreased aconitase activity; increased production of reactive oxygen species; neuroinflammation.
Alvites et al. (2020)	Rat OM-MSCs; karyotype; stemness; secretome; differentiation.
Jafari et al. (2020)	OM-MSCs; adipose tissue (AT) MSCs; comparison; OMSs higher immunosuppressive cytokines.
Tian et al. (2020)	OM-MSCs; Exosome; immunomodulation role; Th1 and Th17 cells differentiation block; inflammatory bowel disease.
Liu et al. (2020)	Cerebral ischemia/reperfusion injury; OM-MSCs; UbiA prenyltransferase domaincontaining 1 (UBIAD1); mitochondrial function; cholesterol metabolism; oxidative/nitrosative stress.
Voronova et al. (2020)	hOM-MSCs; neural lineage; transplantation; spinal cord injuries; hindlimb motor function improvement.
Farhadi et al. (2021)	Undifferentiated hOM-MSCs; transplantation efficacy; PD.
Hamidabadi et al. (2021)	hOM-MSCs; differentiation; motor neuron; spinal cord injury; transplantation.
Zelenova et al. (2021)	Nasal biopsies; ONSs; psychiatric research; neuronal/glial identity.
He et al. (2021)	hOM-MSCs; hypoxia, 1741 lncRNAs differentially expressed;1,603 mRNAs differentially expressed; hypoxia increases proliferation and decreased apoptosis.
Ren et al. (2021)	hOM-MSCs; mouse OMSs; VPA and CHIR99021treatment increases G-protein coupled receptor 5 (Lgr5); generation of sensory neurons generation.
Mollichella et al. (2022)	OM-MSCs; cats; stemness characteristics; multilineage differentiation capacities.
Rövekamp et al. (2022)	OM-MSCs differentiation; neural lineage; transplantation in spinal cord injury efficacy.
Wang et al. (2022)	OM-MSCs; liver protection; plasma aminotransferases enhancement; inflammation reduction; TGF-β decrease; IL-10 increase.
Rantanen et al. (2022)	OM-MSCs; AD; MCI; transcriptome AD and MCI differences.
Entezari et al. (2022)	OM-MSCs; spinal cord injury; OM-MSCs spontaneous differentiation into Schwann cell.
Unzueta-Larrinaga et al. (2023)	Non-invasive exfoliation technique; neurons differentiation.

This table synthesizes outcomes from studies executed between 2011–2023 on olfactory-mucosa–derived stem/progenitor cells, showing consistent feasibility of isolating/expanding cells from humans and multiple species, with cultures displaying neural (Nestin, Sox2, TUJ1, MAP2, GFAP) and often mesenchymal (CD73/CD90/CD105) features and broad multipotency. Many studies demonstrate directed differentiation into neurons, glia, dopaminergic and motor-neuron–like phenotypes, plus mesodermal lineages; functional readouts include electrophysiology, clonal assays, and transplantation with behavioral or physiologic benefit (e.g., improved outcomes in spinal cord injury SCI, Alzheimer’s Disease Parkinson’s Disease (PD), facial nerve palsy (FNP), moderate cognitive impairment (MCI)). Other acronyms: olfactory neurospheres (ONSs); Lamina Propria (LP-MSCs); Olfactory mucosa mesenchymal stem cells (OM-MSCs), human olfactory mucosa mesenchymal stem cells (h OM-MSCs) (cells derived from the olfactory mucosa were indicated as OM-MSCs despite the name that was given in the paper); Globose Basal cells (GBCs); neural stem cells (NSCs); valproic acids (VPA); transforming of growth factor- β (TGF-β); interleukin-10 (IL-10). Ref# = reference number.

**Table 6:** Methods summary.

Study and year	Biopsy	Nasal brush	basic medium	com plex medium	layer	spheres	complex medium/Spheres	complex medium/Layer	basic medium/Spheres	basic medium/Layer	serum free
Benítez-King et al. (2011)		X	X		X					X	
Krolewski et al. (2011)	X			X		X	X				
Toft et al. (2012)	X			X		X	X				
Lindsay et al. (2013)	X			X		X	X				X
Stamegna et al. (2014)	X			X		X	X				X
Thakur et al. (2014)	X			X		X	X				
Ohnishi et al. (2015)	X			X		X					X
Altunbaş et al. (2016)	X		X			X			X		X
Ercolin et al. (2016)	X		X		X					X	
Batioglu-Karaaltin et al. (2016)	X		X		X					X	
Ge et al. (2016)	X			X	X			X			
Tanos et al. (2017)	X		X			X			X		X
Franco et al. (2017)		X		X		X	X				
Lu et al. (2017)	X		X		X						
Muniswami et al. (2017)	X			X		X	X				
Veron et al. (2018)	X			X		X	X				X
Li et al. (2018)	X			X		X	X				X
Jiménez-Vaca et al. (2018)		X		X		X	X				X
Bagher et al. (2018)	X		X		X					X	
Simorgh et al. (2019)	X		X			X			X		
Kim et al. (2019)	X			X		X	X				X
Esaki et al. (2019)	X			X		X	X				X
Salehi et al. (2019)	X		X		X					X	
Alizadeh et al. (2019b)	X		X		X					X	
Bagher et al. (2019)	X		X		X					X	
Alizadeh et al. (2019a)	X		X		X					X	
Solís-Chagoyán et al. (2019)		X	X		X						
Pérez-Luz et al. (2020)	X			X	X			X		X	
Alvites et al. (2020)	X		X		X					X	
Jafari et al. (2020)	X		X		X					X	
Tian et al. (2020)	X		X		X					X	
Liu et al. (2020)	X		X		X					X	
Voronova et al. (2020)	X		X		X					X	X
Farhadi et al. (2021)	X		X		X					X	X
Hamidabadi et al. (2021)	X		X		X					X	
Zelenova et al. (2021)	X		X			X					
He et al. (2021)	X		X		X					X	
Ren et al. (2021)	X			X	X			X			X
Mollichella et al. (2022)	X			X	X			X			X
Rövekamp et al. (2022)	X			X		X	X				
Wang et al. (2022)	X			X	X			X			
Rantanen et al. (2022)	X			X		X	X				X
Entezari et al. (2022)	X			X		X	X				
Unzueta-Larrinaga et al. (2023)		X		X		X	X				X
Count	39	5	22	22	23	21	16	5	3	17	16
Percentage	88,6	11.4	50.0	50.0	52.3	47.7	36,4	11.4	6.8	38.6	36.4

This table summarizes methodological trends in studies isolating olfactory mucosa–derived stem cells across different years and authors. The vast majority of studies (39/44, 88.6%) relied on biopsy as the source of tissue, which varied between the different mammals and mostly did not harm the donor after the extraction, but some animal studies did include killing and decapitation of the participant for the extraction of the sample. Out of the 39 biopsy-based studies, 22 proceeded with basic medium and 22 with complex medium, showing that more than half of the biopsy approaches explored each of these two culture strategies. The minority of studies (5/44, 11.4%) used nasal brush, highlighting biopsy as the dominant approach. Regarding culture conditions, both basic medium and complex medium were equally represented (22/44, 50% each), with a slight preference for layering techniques (23/44, 52.3%) compared to sphere cultures (21/44, 47.7%). When complex medium was combined with either spheres or layering, frequencies were lower (36.4 and 11.4%, respectively), suggesting these are less standard approaches. Basic medium combined with spheres (6.8%) or layering (38.6%) shows that layering is more frequently tested than sphere culture under simpler conditions. Use of serum-free medium was reported in 16 studies (36.4%), which is thought to be safer for clinical use. Overall, the data show a strong reliance on biopsy-based sampling and a relatively balanced exploration of basic vs. complex media, with layering slightly more prevalent than sphere culture, but advanced or combined protocols remain less common. The results mentioned above can be observed in Figure 2.

Variability in medium types also influences outcomes; complex media tend to produce neurospheres (Tables 5, 6; Figure 2; Supplementary Table S1), whereas basic media often result in monolayer cultures, highlighting the role of environmental factors in OM-MSC growth and differentiation. This review confirms that studies have been able to isolate stem cells from the olfactory mucosa of humans and other mammals successfully, and highlights the appropriate collection, isolation, characterization, storage, and experimental application methods for this process of isolation (Table 7).

**Table 7:** Quantitative trends and simplified quality appraisal across included studies.

Domain	Category	*n*/N (%)	Interpretation/Implication
Sampling	Biopsy	39/44 (88.6%)	Provides deeper lamina propria tissue and higher likelihood of capturing OM-MSCs, invasive.
Sampling	Brushing	5/44 (11.4%)	Less invasive, may preferentially sample epithelial cells, thus lead to lower standardization.
Culture medium	Basic vs. complex	22/44 vs. 22/44 (50% each)	Medium composition strongly associated with monolayer vs. sphere growth.
Culture format	Adherent vs. spheres	23/44 vs. 21/44 (52.3% vs. 47.7%)	Adherent cultures predominate slightly, spheres more common with complex media.
Clinical translation	Serum-free reported	16/44 (36.4%)	Important for GMP/clinical compliance, still underused in the field.

OM-MSCs hold promise as a model for researching neural development (Benítez-King et al., 2011) and as a brain progenitor cell source for characterization from physiological and molecular perspectives (Thakur et al., 2014; Ohnishi et al., 2015; Ge et al., 2016; Tanos et al., 2017; Franco et al., 2017; Jiménez-Vaca et al., 2018; Solís-Chagoyán et al., 2019; Alvites et al., 2020). After their complete characterization, a preclinical approach was performed in many different studies in order to assess their utility to treat different diseases in animal models (mostly rodents). These investigations have included models of peripheral nerve injury (Batioglu-Karaaltin et al., 2016; Entezari et al., 2022), neuropsychiatric disorders such as bipolar disorder and schizophrenia (Benítez-King et al., 2011), retinal disease (Lu et al., 2017), olfactory dysfunction (Li et al., 2018; Ren et al., 2021), nerve palsy (Esaki et al., 2019), Parkinson’s disease (Alizadeh et al., 2019a), motor neuron disease models (Bagher et al., 2019), spinal cord injury (Voronova et al., 2020; Bagher et al., 2018), inflammatory conditions (Tian et al., 2020), and autologous grafting approaches (Muniswami et al., 2017). Importantly, these studies remain exploratory and are intended to evaluate cellular properties and experimental feasibility rather than to demonstrate therapeutic efficacy.

Despite their potential, several challenges need to be addressed for the clinical translation of OM-MSCs. Variations in isolation techniques, donor heterogeneity, and culture conditions contribute to inconsistencies in outcomes across studies. For instance, differences between biopsy methods (e.g., nasal brush versus surgical biopsy) and culture media (e.g., basic versus complex) can significantly influence the yield and quality of OM-MSCs. Additionally, ethical concerns and potential risks associated with invasive biopsy techniques must be mitigated through advancements in non-invasive collection methods.

Biopsy vs. Brushing: Biopsy has been shown to be the preferred method for practical and biological reasons. Technically, biopsy provides a larger and deeper fragment which contains lamina propria, the compartment most consistently reported to harbor mesenchymal-like OM-MSC/OE-MSC populations. This increases the probability of obtaining fibroblast-like adherent colonies and of recovering cells expressing canonical MSC markers (CD73/CD90/CD105) alongside neural markers (e.g., Nestin/Sox2). In contrast, brushing collects superficial epithelial cells and debris, and sampling depth is operator-dependent; consequently, brushing may demonstrate an increased amount of epithelial progenitors or mixed epithelial–neuronal cells rather than mesenchymal stromal populations, potentially explaining lower adoption and more variable yields. From a workflow standpoint, biopsy specimens allow cleaner mechanical mincing and controlled enzymatic digestion, while brush samples often require additional washing/filtration steps and may carry a higher microbial load. Clinically, brushing has clear advantages (low invasiveness, repeatability, suitability for longitudinal sampling), but the field lacks standardized brush type, number of rotations, anatomical site, and post-collection processing—limiting reproducibility. Future work should focus on protocol harmonization for brushing (site, depth surrogate, processing, and marker panels) to enable it as a clinically scalable alternative to biopsy.

However, the use of adult stem cells with respect to embryonic stem cells has much less ethical implication. First, because the tissue of origin is an adult tissue, and its collection does not cause the loss of the embryo, second, because this tissue can be harvested by means of non-invasive methods such as brushing, or it can be obtained by means of biopsy during surgery with little impact for the patients.

Indeed, all the human studies, we analyzed in this review necessitated, just the written informed consent prior to their involvement in the study, which was previously approved by the institution’s ethics committee (Benítez-King et al., 2011; Lindsay et al., 2013; Ohnishi et al., 2015; Batioglu-Karaaltin et al., 2016; Ge et al., 2016; Tanos et al., 2017; Franco et al., 2017; Lu et al., 2017; Jiménez-Vaca et al., 2018; Bagher et al., 2018, 2019; Alizadeh et al., 2019a, 2019b; Solís-Chagoyán et al., 2019; Pérez-Luz et al., 2020; Jafari et al., 2020; Liu et al., 2020; Voronova et al., 2020; Farhadi et al., 2021; Hamidabadi et al., 2021; Zelenova et al., 2021; He et al., 2021; Ren et al., 2021; Rövekamp et al., 2022; Rantanen et al., 2022; Unzueta-Larrinaga et al., 2023; Bredenoord et al., 2017; King and Perrin, 2014).

Even if the ethical issues relative to the harvesting of olfactory mucosa mesenchymal stem cells (OM-MSCs) are minimal, especially in comparison to embryonic stem cells, numerous *in vitro* and preclinical results have not always been satisfactory. There is great hope for desperate patients looking for a real cure for many diseases with poor prognoses and incurable conditions, but many businesses and technological companies in the stem cell industry failed to produce a convincing, reproducible, FDA-licensed, and effective stem cell remedy. The challenges of translating preclinical findings into clinical outcomes are common in many pharmacological research domains and are not unique to stem cell therapy. According to statistics, over 90% of Phase III clinical studies in the pharmaceutical industry do not result in a novel medication that is commercially available. As of right now, this parallel highlights that most of the stem cell therapies face the same difficulties as other medical treatments, such as issues with clinical effectiveness, safety worries, and regulatory approval (International Society for Stem Cell Research (ISSCR), 2019) with the exception of mesenchymal stem cells extracted from the bone marrow of humans (Marei, 2025; Thompson et al., 2020).

Another important ethical point regarding the transplantation of the stem cells includes the tissue of origin that cannot be verified in terms of diseases of the donor or the methods of cultivation that could be contaminated by animal components. This is particularly frequent in cells offered by unverified clinics, and for this reason patients must be aware of the need for authorizations to the clinics by regulatory agencies FDA and EMA (International Society for Stem Cell Research (ISSCR), 2019).

In order for stem cell treatments to fulfil their potential, reproducibility concerns must be resolved, and they need to be standardized. Numerous illnesses, such as neurological disorders, diabetes, heart issues, and some forms of cancer, may be effectively treated using stem cell treatment. The development of stem cell-based therapies from the lab to the clinical setting has been impeded by issues with reproducibility and standardization, which are the main reasons why it is still so difficult to get consistent and trustworthy results in various labs and clinical settings. The International Society for Stem Cell Research (International Society for Stem Cell Research (ISSCR), 2019) estimates that there are between 700 and 1,000 uncontrolled stem cell clinics operating worldwide, primarily in countries with inadequate control, such as the United States, Mexico, Eastern Europe, and Thailand. Since these clinics promise quick access to treatment, patients are typically drawn from countries with strict laws, making law enforcement and control difficult. Even in countries with strict regulations on stem cell therapies, patients may travel abroad to receive illegal treatments, complicating efforts to ensure their safety (Marei, 2025; Thompson et al., 2020; Barmada et al., 2023).

### Strengths and limitations

#### Strengths

This review conducted a comprehensive search across three databases using a PRISMA-guided selection process. It included both human and multi-species studies to capture the breadth of available protocols. Data extraction was structured and systematic, covering key methodological domains such as sampling techniques, processing workflows, culture conditions, and marker panels. In addition, the study provided a quantitative synthesis to describe major methodological trends across the literature.

#### Limitations

Interpretation was limited by high methodological heterogeneity and inconsistent reporting across studies, which prevented a formal meta-analysis. Variability in terminology and marker panels likely reflects protocol-dependent phenotypes, reducing comparability between datasets. The limited number of brushing-based studies further constrained cross-study comparisons. Finally, most included studies were preclinical or *in vitro* and often provided incomplete reporting of bias-control measures, such as randomization and blinding in animal experiments.

## Conclusion

This systematic review allows us to support our idea that olfactory mucosa could be a good source of material to produce cells with neuroectodermal features. These cells could represent a valid alternative to other types of cells for neurological applications.

The body of research on OM-MSCs underscores their immense potential in regenerative medicine, particularly for neurodegenerative and immune-mediated conditions. Their unique combination of neural, glial, and mesenchymal characteristics, coupled with their ability to secrete neurotrophic and immunomodulatory factors, makes them a versatile and promising cell source. Preclinical and clinical studies have demonstrated their efficacy in promoting functional recovery and tissue repair, especially in neurological applications.

However, to clinically implement these findings it is important to address key challenges, including standardizing isolation and culture protocols, ensuring safety and reproducibility, and navigating regulatory hurdles. As research continues to refine the understanding of OM-MSC biology and therapeutic mechanisms, their integration into mainstream clinical practice appears increasingly feasible. Collaborative efforts between researchers, clinicians, and regulatory bodies will be crucial in unlocking the full therapeutic potential of OM-MSCs, paving the way for innovative and effective treatments for a broad spectrum of diseases.

### Future directions

To accelerate translation, future studies should adopt a standardized reporting of sampling site and depth (including whether lamina propria is captured). Along with harmonize minimal marker panels distinguishing epithelial NSCs from OM-MSCs (MSC markers + neural markers + exclusion of epithelial cytokeratins), directly compare brushing vs. biopsy within the same donors using predefined brush devices and sampling metrics, move toward xeno-free/serum-free and GMP-compatible media, and include functional potency assays (secretome profiling, immunomodulation, and standardized neuronal differentiation endpoints). Multicenter protocol-consensus studies would be the most efficient approach to identify the most reproducible and scalable isolation strategy.

These findings reinforce the notion that OM-MSCs represent a groundbreaking avenue for therapeutic innovation, with the potential to transform the landscape of regenerative medicine. By addressing the remaining challenges through continued research and interdisciplinary collaboration, OM-MSCs could pave the way for advanced treatments that significantly improve patient outcomes across a wide range of neurological and immune-mediated conditions.
