# Supplementary material for: Systematic review of literature regarding the isolation of mesenchymal adult stem cells from the olfactory epithelium
Source: Front Cell Neurosci. 2026 Mar 31;20:1735284. doi: 10.3389/fncel.2026.1735284 (PMC13076147; doi:10.3389/fncel.2026.1735284)
Supplement: Supplementary file 1 [file Table_1.docx]

Supplementary Data

Fig 1S

| **Ref #** | **Study Author** | **Results of the Analysis** |
| --- | --- | --- |
| 26 | Benítez-King et al, 2011 | 1. Exfoliated cells from the nasal brush were expanded and a neural precursor bank was built. 2. Thawed cells displayed neuronal developmental cytoskeletal abnormalities. |
| 27 | Krolewski et al, 2011 | 1. The main results is how the cell fate within ONSs is impacted by conditioned media obtained from lamina propria derived cells. 2. They also included studies that aim to provide an extra rigorous test for the in vitro model's biological relevance by comparing results from a transplantation assay with findings from the ONS system. |
| 28 | Toft et al, 2012 | 1. This study demonstrates that olfactory ensheathing cells derived from adult olfactory tissue exhibit no advantages over undifferentiated olfactory spheres, despite the latter's capacity to facilitate axonal regeneration. 2. Compared to pure olfactory ensheathing cells, olfactory spheres caused a larger astrocytic hypertrophy at the damage site. |
| 29 | Lindsay et al, 2013 | 1. LP-MSCs may improve oligodendrocyte myelination in a well-established in vitro myelinating culture method and change the biological characteristics of OECs and oligodendrocyte precursor cells (OPCs) in vitro. |
| 30 | Stamegna et al, 2014 | 1. Process that allows the generation of rat nasal cells. |
| 31 | Thakur et al, 2014 | 1. GBCs were discovered to be positive for all markers of neural progenitor cells and could be extracted in pure (99% purity) form. 2. The absence of any voltage-gated Na+ channels indicates that GBCs are unexcitable. On the GBC, leaky K+ channels were discovered, however this was not significant. |
| 32 | Ohnishi et al, 2015 | 1. The results of this investigation imply that human OS cells may self-differentiate into neurons in culture environment and may be utilized as a source of neural progenitors for self-regenerative grafts, circumventing the requirement for immunosuppression and morally dubious situations. |
| 33 | Altunbaş et al, 2015 | 1. The objective was to separate and characterize a canine OM stem cell population as a substitute for bone marrow and adipose tissue in the production of adult stem cells. |
| 34 | Ercolin et al, 2016 | 1. The OSCs had mesenchymal characteristics, including as plasticity and a positive reaction to the antibodies CD34, CD73, and CD90. 2. These cells also multiply quickly and can be maintained in culture through many passages, allowing them to continue proliferating and differentiating even after being cryopreserved. 3. Cellular tracking in vivo is made possible by the positive transduction response. |
| 35 | Batioglu-Karaaltin et al, 2016 | 1. Human subjects’ olfactory mucosa was used to extract olfactory stem cells. 2. Rats receiving these cells as a transplant showed considerably higher whisker-movement scores. |
| 36 | Ge et al, 2016 | 1. In this work, SDS-PAGE and LC-MS were used to examine the secretome of OM-MSCs. 2. There were found to be 274 secreted proteins. 3. Given that these chemicals are known to have a substantial role in neurotrophy, angiogenesis, cell proliferation, differentiation, apoptosis, and inflammation, OM-MSC transplantation may considerably enhance CNS healing. |
| 37 | Tanos et al, 2017 | 1. This study described the production of primary cell cultures from human OM biopsies found in the olfactory mucosa (OM) of adult patients and those obtained from OM cell cultures. 2. It describes also the isolation of putative OM stem cells identified by the retention of the fluorescent dye known as PKH26. |
| 38 | Franco et al, 2017 | 1. According to this study, human nasal olfactory stem/progenitors cells and mice share pluripotency-related gene expression. |
| 39 | Lu et al, 2017 | 1. Human olfactory mucosa mesenchymal stem cells (hOM)-MSCs were based on spindle cells and exhibited radial colony organization. OM-MSCs were shown to be positive for CD73 and CD90 but negative for CD34, CD45, and CD105. 2. After induction, the cells' production of the photoreceptor-specific marker rhodopsin resulted in a robust positive response. |
| 40 | Muniswami et al, 2017 | 1. GBCs expressed neural stem cell markers and mesenchymal stem cell markers, they can be differentiated in cells expressed neuronal markers. |
| 41 | Veron et al, 2018 | 1. Olfactory mucosa explants can be used to quickly produce cell populations. 2. Cells shared important characteristics with human counterparts, including a fibroblastic shape, strong nestin expression, sphere-forming capacity, and comparable surface marker expression (CD44, CD73). 3. Majority of them demonstrated clonogenicity with genus-specific characteristics and significant rates of proliferation. 4. The capacity to develop into mesodermal lineages was also demonstrated by OE-MSCs. |
| 42 | Li et al, 2018 | 1. Olfactory neuroepithelial cells create olfactory neurospheres on chitosan films with continuously increasing diameter, in contrast to the flat layer on controls. 2. Both immature and adult olfactory receptor neurons are found in the olfactory neurospheres, along with basal cells. 3. Olfactory marker protein expression is greater on chitosan films compared to controls in terms of gene and protein levels, and a similar tendency is also expressed by the olfactory transduction components. |
| 43 | Jiménez-Vaca et al, 2018 | 1. Olfactory neuroepithelium exfoliation was used to produce stem cells that was demonstrated to be of neural origin. |
| 44 | Bagher et al, 2018 | 1. Mesenchymal stem cells from olfactory mucosa are abundant and available stem cells. The olfactory mucosa stem cells have tendency to differentiate into motor neurons. |
| 45 | Simorgh et al, 2019 | 1. Using a rat model of Parkinson's disease, the therapeutic potential of OEMSCs was assessed by transplanting isolated cells. 2. The OEMSCs caused a notable improvement in the behavior of Parkinsonian rats. In comparison to the control group that is investigated, the results show that OEMSCs raised the expression of PAX2, PAX5, PITX3, dopamine transporter, and tyrosine hydroxylase of these proteins. 3. The implantation of fully developed OE-MSCs into dopaminergic neurons has been shown to significantly improve Parkinson's patients' condition. |
| 46 | Kim et al, 2019 | 1. Neural stem cell markers are expressed by OSCs. By means of immunocytochemical staining neuronal differentiation was studied and discovered that OSCs had considerably higher expressions of Tuji1, GFAP, and O4. 2. Allergic rhinitis model had considerably fewer neurospheres, a lower potential for clonogenic growth, and lower expression of markers for olfactory NSCs. |
| 47 | Esaki et al, 2019 | 1. Assessment of utility of OSC transplantation in vivo as a treatment for facial nerve palsy in a mouse model 2. When OSCs and hydrogel were combined, the effects of the rapid recovery from facial nerve palsy were much more apparent and improved during the course of the research. |
| 48 | Salehi et al, 2019 | 1. Assessment of efficacy of peripheral nerve regeneration using OE-MSCs carried by alginate/chitosan (alg/chit) hydrogel in a rat sciatic nerve defect. 2. Alg/chit hydrogel with OE-MSCs to the sciatic nerve defect enhances regeneration compared to the control group and hydrogel without cells, according to the results of the sciatic functional index, hot plate latency, electrophysiological assessment, weight-loss percentage of wet gastrocnemius muscle. |
| 49 | Alizadeh et al, 2019 | 1. This study demonstrated that 12 days after differentiation, differentiated OE-MSCs may exhibit considerable DA neuron markers expression at the mRNA and protein levels in addition to dopamine release. |
| 50 | Bagher et al, 2019 | 1. The findings of this study imply that OE-MSCs on conductive hydrogels might successfully develop into cells resembling motor neurons. |
| 51 | Alizadeh et al, 2019 | 1. While WJ-MSCs are separated from the Wharton's jelly area of the umbilical cord, which is seldom accessible to all adults, OE-MSCs may be readily extracted from the adult human nasal cavity. 2. The OE-MSCs have a greater ability for proliferation than the WJ-MSCs while expressing the same mesenchymal markers. 3. When compared to WJ-MSCs, the OE-MSCs demonstrated a higher capacity for differentiation into dopaminergic neurons. Nearly the same quantity of dopamine was released by dopaminergic neurons that were differentiated from both cell types, indicating a very slight change in their functioning. 4. When OE-MSCs are compared to WJ-MSCs, their superior harvestability, high rate of proliferation, and capacity for autologous transplantation indicate that they are a superior cell source. |
| 52 | Solís-Chagoyán et al, 2019 | 1. MpSC-like hONPCs with ex vivo-expressed functional P2 receptors control exocytosis and Ca2+ signaling. 2. Paracrine signaling among hOE cells may be mediated by this release of biochemical messengers to the extracellular environment, which is induced by purines. |
| 53 | Pérez-Luz et al, 2020 | 1. Human olfactory ecto-mesenchymal stem cells from Friedreich's ataxia patients and healthy donors were isolated and cultured for this study. 2. The cells' phenotype was described, along with disease-specific characteristics like decreased cell viability, decreased aconitase activity, increased production of reactive oxygen species, and the release of cytokines linked to neuroinflammation. 3. Benefit on cells generated from patients when frataxin levels were raised, indicating that this in vitro model is useful for researching the illness. |
| 54 | Alvites et al, 2020 | 1. Rat OM-MSCs are fully characterized utilizing a sequential, longitudinal methodology not seen in previous research. 2. Protocols for freezing and thawing cells, as well as techniques for cell separation, culture, and growth, received particular attention. 3. The biological characterization involved analyzing the behavior of the cells in culture, identifying specific proteins from the cell surface, determining the cellular karyotype, testing the capacity for multilineage differentiation, characterizing the secretome profile during expansion, and assessing the immune-modulatory capacity. 4. Additionally, the OM-MSC stemness features were assessed in anticipation of their potential application in regenerative treatments. |
| 55 | Jafari et al, 2020 | 1. According to this study, OE-MSCs have a stronger proliferation capacity than ATMSCs. 2. TLR3 and TLR4 gene expression is higher in OE-MSCs than in AT-MSCs.But TLR3 expression by OE-MSCs appears to be nonfunctional, in contrast to AT-MSCs. According to the research, OE-MSCs secrete more immunosuppressive cytokines than AT-MSCs. OE-MSCs are among the finest MSC sources for autoimmune disease cell treatment as well as regenerative medicine. |
| 56 | Tian et al, 2020 | 1. The immunomodulatory role of OE-MSCs-Exos in controlling T-cell responses was examined in this work. The immune-suppressive ability of OE-MSCs-Exos was demonstrated by blocking the differentiation of Th1 and Th17 cells and encouraging the production of regulatory T (Treg) cells. 2. OE-MSCs-Exos were shown to lessen the severity of the disease in vivo by employing the experimental colitis mice model. This was followed by a rise in Treg cells and a decrease in Th1/Th17 cell responses. |
| 57 | Liu et al, 2020 | 1. The significance of UBIAD1 in mitochondrial function, cholesterol metabolism, and oxidative/nitrosative stress is highlighted by these investigations. 2. Protective effects of OM-MSCs in cerebral ischemia/reperfusion injury using the oxygen-glucose deprivation/reperfusion (OGD/R) model in vitro and the transient middle cerebral artery occlusion (t-MCAO) model in vivo. 3. It also examined whether OM-MSCs protect neurons by reducing mitochondrial dysfunction and boosting antioxidant activity through upregulation of UBIAD1. |
| 58 | Voronova et al, 2020 | 1. This study examined the effectiveness of using human olfactory mucosa neural stem/progenitor cell transplantation for persistent spinal cord injuries. 2. Development of a methodology to harvest neural stem/progenitor cells, given to rats suffering from spinal post-traumatic cysts. It was demonstrated that in recipient rats with spinal post-traumatic cysts transplantation of neural stem/progenitor cells from human olfactory lining enhanced hindlimb motor function. |
| 59 | Farhadi et al, 2021 | 1. Results suggest that undifferentiated OE-MSCs might be considered a suitable source for cell replacement treatment, specifically targeted at Parkinson's disease. |
| 60 | Hamidabadi et al, 2021 | 1. Potential use of motor neuron-like cells from hOE-MSCs as a different cell-based therapy approach for spinal cord damage. |
| 61 | Zelenova et al, 2021 | 1. The molecular alterations that take place during the transition from neurospheres (NS) to neurospheres-derived cells (NDC) from nasal biopsies were investigated using differential expression analysis. 2. It was discovered that NDCs had downregulated pathways linked to vascular and neural cells. NDC was shown to have a reduction in neuronal and glial components when compared to public transcriptomes. 3. NDC had a number of metabolic traits unique to neural progenitors that were given the fungicide maneb (manganese ethylene-bis-dithiocarbamate). 4. Technique modifications are required to employ NDC for bulk sample collection in psychiatric research, even if NDC retains some neuronal/glial identity. |
| 62 | He et al, 2021 | 1. A total of 1741 lncRNAs and 1603 mRNAs were found to be significantly differently expressed in the hypoxia group compared with the normoxia group by comparative analysis and bioinformatics analysis. Enrichment analysis showed that the genes of human OM-MSCs with variable expression were mostly involved in the control of the cell cycle, cytokine secretion, and other processes. 2. Hypoxic conditions increased the proliferation and decreased the apoptotic response of human OM-MSCs. |
| 63 | Ren et al, 2021 | 1. VPA and CHIR99021 caused the highest amount of Lgr5 expression in specified culture medium, but in mouse colonies, LY411575 produced the most plentiful supply of OMP+ mature sensory neurons. 2. A morphological shift from a filled to a cystic look was seen in different base culture medium with drug combinations, which was followed by significant transcriptional alterations in several signaling pathways. 3. In human colonies, TGF-β signaling influenced the generation of sensory neurons, whereas VPA controlled the expression of Lgr5 and the proliferation of cells. |
| 64 | Mollichella et al, 2022 | 1. For the first time, the study demonstrates that it is feasible to separate OE-MSCs from the olfactory mucosa of cats. 2. These cells demonstrated multilineage differentiation capacities and stemness characteristics. |
| 65 | Rövekamp et al, 2022 | 1. This study showed that OSCs differentiated into the neural lineage and expressed both b-III tubulin and GFAP. In addition, OSCs multiplied and were viable in a matrix of plasma clots. |
| 66 | Wang et al, 2022 | 1. This study demonstrates that the lysate of OM-MSCs may successfully protect the liver, enhance plasma aminotransferases, and reduce inflammation through the transformation of growth factor-beta (TGF-β) and the release of interleukin-10 (IL-10). |
| 67 | Rantanen et al, 2022 | 1. Human olfactory mucosal biopsies from age-matched cognitively healthy adults (HC), patients with LOAD (AD), and people with moderate cognitive impairment (MCI), a prodromal AD condition, were used to create human olfactory neurosphere-derived (ONS) cell cultures in this work. 2. The authors looked at the entire transcriptome of ONS cells and the development properties of olfactory neurospheres to identify variations in gene expression in AD and MCI patients. |
| 68 | Entezari et al, 2022 | 1. The results showed that OE-MSCs could develop into phenotypes like those of Spinal cord on their own. |
| 69 | Unzueta-Larrinaga et al, 2023 | 1. This study describes how to isolate and cultivate human differentiated neurons and glial cells from live participants' olfactory epithelium using a simple, non-invasive exfoliation technique. |

**Legend Table 1S: Results of the Analysis of the Culture derived Cells.**

Table 1S synthesizes outcomes from studies executed between 2011–2023 on olfactory-mucosa–derived stem/progenitor cells, showing consistent feasibility of isolating/expanding cells from humans and multiple species, with cultures displaying neural (Nestin, Sox2, TUJ1, MAP2, GFAP) and often mesenchymal (CD73/CD90/CD105) features and broad multipotency. Many studies demonstrate directed differentiation into neurons, glia, dopaminergic and motor-neuron–like phenotypes, plus mesodermal lineages; functional readouts include electrophysiology, clonal assays, and transplantation with behavioral or physiologic benefit (e.g., improved outcomes in spinal cord injury, Parkinson’s models, facial nerve palsy). Ref#= reference number.
